# Case Report: *Mycoplasma pneumoniae*–associated acute pancreatitis

**DOI:** 10.3389/fped.2024.1416189

**Published:** 2024-09-04

**Authors:** Hong Sun, Wei-Qun Wang, Long Lin, Zheng-Yang Shao, Lu Zhan, Lan-Fang Tang

**Affiliations:** ^1^ Department of Pediatrics, Zhejiang Hospital of Integrated Traditional Chinese and Western Medicine, Hangzhou, Zhejiang, China; ^2^ Department of Pulmonology, Children’s Hospital of Zhejiang University School of Medicine, Hangzhou, Zhejiang, China

**Keywords:** *Mycoplasma pneumoniae*, extrapulmonary complications, acute pancreatitis, cytokine, children

## Abstract

*Mycoplasma pneumoniae* is the primary pathogen causing community-acquired pneumonia in children, accounting for approximately 10%–40% of cases. It can lead to various extrapulmonary complications, including acute pancreatitis, which has been reported in approximately 30 cases to date. Here, we report a 4-year-old girl who presented with fever, cough, and elevated levels of *M. pneumoniae* IgM antibodies, followed by the onset of abdominal pain, elevated lipase, and elevated blood and urine amylase. Abdominal CT implied diffuse inflammation of the pancreas. Serum inflammatory cytokines, such as interleukin (IL)-2, IL-6, IL-17A, tumor necrosis factor, and interferon-gamma, were elevated. After excluding other causes, it was determined that *M. pneumoniae* infection was the cause of her acute pancreatitis. She was treated with macrolides and glucocorticoids and ultimately made a full recovery. Therefore, acute pancreatitis should be included in the differential diagnosis for patients with *M. pneumoniae* infection who present with abdominal pain. Inflammatory cytokines may play a role in mediating pancreatic damage.

## Introduction

*Mycoplasma pneumoniae* (MP) is the primary pathogen causing community-acquired pneumonia in children, accounting for approximately 10%–40% (1, 2). It can lead to various extrapulmonary complications, including dermatological, nervous system, hematologic, cardiovascular, and gastrointestinal damage. Acute pancreatitis (AP) is a rare complication that has occasionally occurred since 1973 (3). To date, there are only about 30 relevant reports. Therefore, it is often overlooked, and the pathogenesis remains unclear. Several patients have been misdiagnosed as having appendicitis and have undergone appendectomy.

Consequently, when children with *M. pneumoniae* pneumonia (MPP) experience abdominal pain, special attention should be paid, more thinking should be made, and various possibilities should be investigated. Rare complications such as AP should not be disregarded. Here, we report a 4-year-old girl who developed AP due to MP infection, accompanied by disordered serum cytokine levels and immune function, which suggests that she may have had an underlying immune disorder.

## Case presentation

A 4-year-old girl presented with a cough for 10 days and a fever for 4 days. Her cough was mild for the first 6 days. Then, on the sixth day after the onset of the disease, it became severe, scoring 7 on the visual analog scale (VAS). Concurrently, she had a fever with a peak temperature of 39.2℃. She did not report any other symptoms. At that time, her neighborhood was experiencing an MPP outbreak. A local hospital doctor prescribed her oral azithromycin for 3 days, but her body temperature remained between 38℃ and 39℃, and the cough score had not decreased. She vomited once before coming to our hospital.

She had a history of bilateral renal dysplasia, vesicoureteral reflux, atrial septal defect, and Zhu–Tokita–Takenouchi–Kim syndrome, but no congenital abnormalities of the pancreaticobiliary system were noted. Her kidney function was normal. She had no history of infectious diseases or exposure to infected individuals. Her parents confirmed that she had no contact with sick or dead poultry. She had a history of positive skin tests for penicillin and ceftriaxone, indicating an allergy. She was allergic to formula milk and had no reported history of allergies to other foods or medications. She was born full-term via cesarean section without any history of birth injury or asphyxia; her birth weight was approximately 2.5 kg. Her physical development, particularly in sports, was delayed. Her father had a history of allergic rhinitis and her mother was in good health. Her parents reported no family history of hereditary diseases.

The physical examination revealed a height of 96 cm, a weight of 13 kg, a body temperature of 37.7℃, a respiratory rate of 26 beats per minute, and a blood pressure of 98/54 mmHg. Pharyngeal congestion, grade I enlargement of both tonsils without exudation, slight dry rales in both lungs, and cardiac auscultation showed no abnormalities. Her abdomen was flat and soft, without tenderness, and no hepatosplenomegaly was detected.

**Figure 1:**
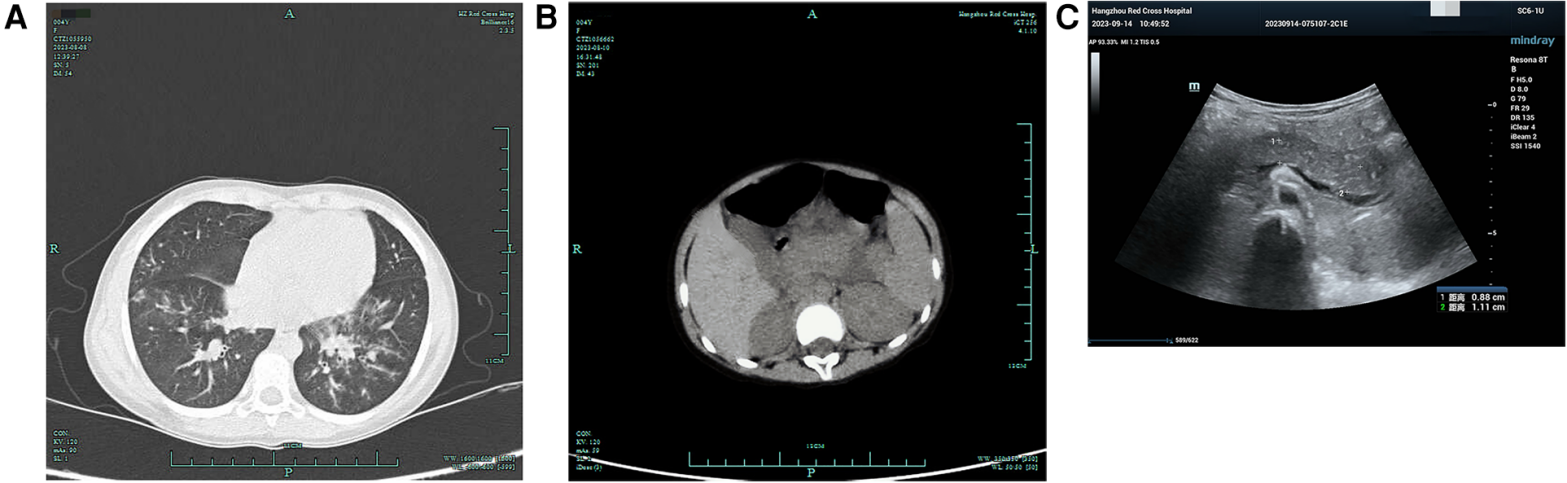
Lung and pancreas images. **(A)** Lung CT showed scattered patchy high-density blurred shadows in both lungs, without pleural effusion and thickened pleura. **(B)** Abdominal CT showed pancreatic enlargement with poor visualization, blurred surrounding spaces, and occasional fluid accumulation shadows. **(C)** Abdominal ultrasound showed the pancreatic head, body, and tail measured approximately 1.7, 1.4, and 1.3 cm in thickness, respectively. There was a significant fluid accumulation of approximately 1.0 cm in the intestinal gap. Fluid accumulation could be seen in the liver and kidney crypts, as well as the spleen and kidney crypts, with a maximum depth of approximately 0.7 cm.

At the time of admission, initial laboratory tests revealed a hemoglobin of 10.5 g/dl, a white blood cell count of 3,800/mm^3^, comprising 55.7% neutrophils and 37.0% lymphocytes, and a platelet count of 207 × 10^3^/mm^3^. C-reactive protein was 6.09 mg/L. Electrolyte and biochemistry laboratory tests showed that the levels of amylase, lipase, alkaline phosphatase, lactate dehydrogenase, triglyceride, and blood glucose were within the normal range. Polymerase chain reaction (PCR) for respiratory viruses, including adenovirus, influenza, respiratory syncytial virus, and coronavirus, was negative, as was sputum culture. Deep sputum aspiration was performed to detect MP nucleic acid, and the result was positive. The IgM antibody for MP, measured by chemiluminescence, was positive (1.13 COI, reference range ≤1.10 COI). She was treated with intravenous erythromycin for the infection. On the second day after admission, her body temperature remained high, and lung computed tomography (CT) showed scattered, patchy, high-density blurred shadows in both lungs, without thick pleural or pleural effusion observed in the chest cavity (Figure 1A). Therefore, she was given anti-inflammatory and immunomodulatory treatment with methylprednisolone sodium succinate. Unfortunately, after 1 day of treatment with methylprednisolone sodium succinate (the third day after admission), her blood glucose level rose to 15 mmol/L and her urine glucose test was positive. Therefore, the administration of methylprednisolone sodium succinate was discontinued. Subsequently, her blood glucose level gradually returned to normal.

She developed abdominal pain on the fourth day after admission without vomiting, diarrhea, dyspnea, or tachypnea. Physical examination showed tenderness in the left upper abdomen accompanied by mild abdominal muscle tension. Laboratory studies showed a serum lipase of 1,193 U/L (normal range, <15–116 U/L) and amylase of 625 U/L (normal range, 13–60 U/L). Meanwhile, urinary amylase also increased to 8,827 U/L (normal range, <750 U/L). Abdominal ultrasound and abdominal CT showed ascites and enlargement of the pancreas (Figures 1B,C).

No abnormalities of the liver or biliary system were noted. Fasting blood glucose, insulin, and glycated hemoglobin were all within the normal range. Laboratory tests revealed a hemoglobin of 11.3 g/dl, a white blood cell count of 5,800/mm^3^, comprising 72.8% neutrophils and 19.7% lymphocytes, a platelet count of 298 × 10^3^/mm^3^, and C-reactive protein of 2.92 mg/L. Serum levels of interleukin (IL)-2, IL-6, IL-17A, tumor necrosis factor (TNF), and interferon-gamma (IFN-γ), detected using a chemiluminescence method, were significantly increased (Table 1). The patient was immediately fasted and treated with an intravenous infusion. She received total intravenous nutrition and omeprazole treatment. She received treatment with methylprednisolone again, and this time, her blood sugar levels were normal. Three days later (the seventh day after admission), her abdominal pain symptoms significantly improved and she began to eat. The inflammatory cytokines, blood, and urine amylase levels gradually decreased (Table 1). Therefore, the increase in blood sugar after the initial use of methylprednisolone was due to stress-induced hyperglycemia. After using methylprednisolone again, rigorous blood sugar monitoring was conducted and found to be within the normal range.

**Table 1:** Changes in cytokines, lipase, and amylase after acute pancreatitis.

	Day 1	Day 2	Day 3	Day 4	Day 14
CRP (normal range, 0.00–3.30 mg/L)	2.92	—	—	0.63	—
WBC (normal range, 3,500–9,500/mm^3^)	5,800	–	—	8,100	—
IL-2 (normal range, ≤5.71 pg/ml)	108.42	—	—	—	38.37
IL-6 (normal range, ≤5.3 pg/ml)	96.95	—	—	—	21.97
IL-17 (normal range, ≤20.6 pg/ml)	24.11	—	—	—	8.95
IFN-γ (normal range, ≤7.42 pg/ml)	62.87	—	—	—	7.13
TNF (normal range, ≤4.6 pg/ml)	107.81	—	—	—	31.47
Blood amylase (normal range, 15–116 U/L)	625	300	106	113	—
Urine amylase (normal range, <750 U/L)	8,827	2,687	1,312	564	—
Lipase (normal range, 13–60 U/L)	1,193	222	85	93	—

CRP, C-reactive protein; WBC, white blood cell.

**Figure 2:**
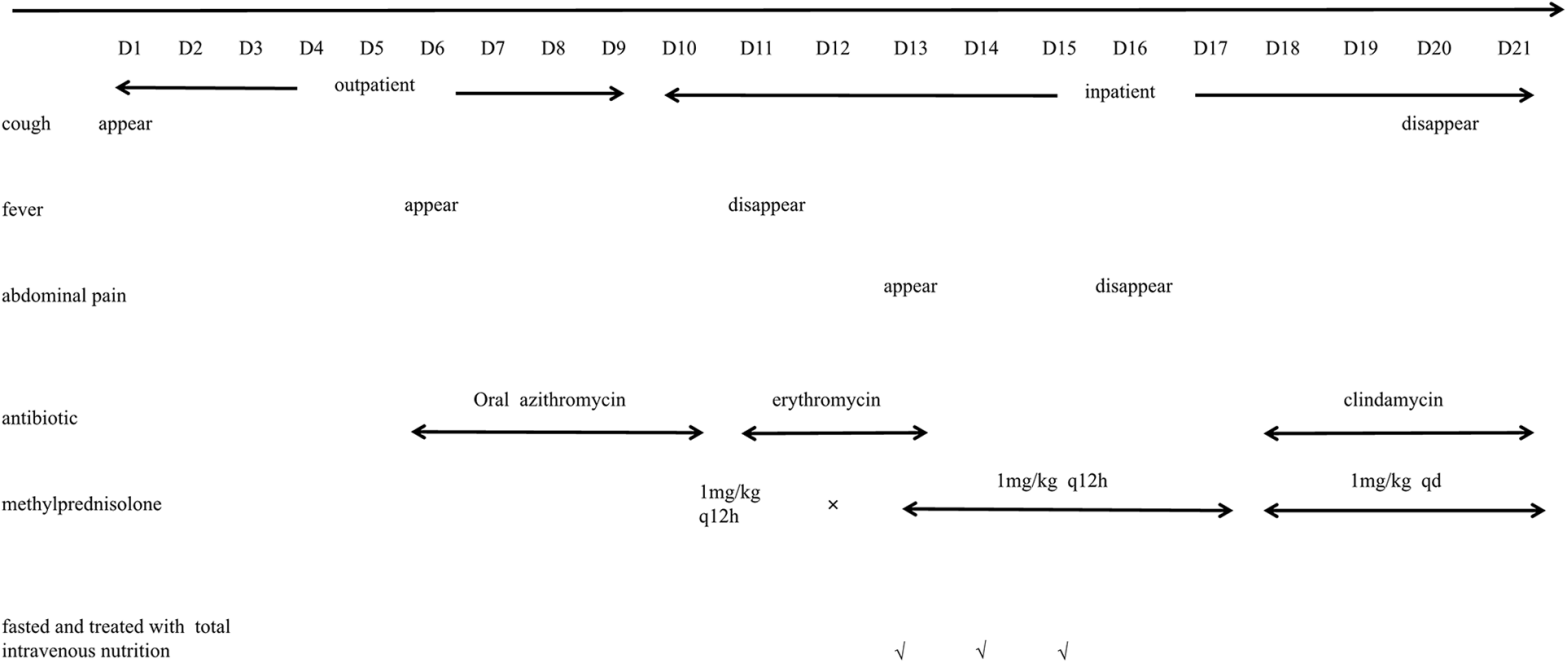
Timeline of the disease process.

Interestingly, when she underwent an abdominal ultrasound examination 6 days after the onset of pancreatitis (the 10th day after admission), the intense echogenicity of the gallbladder changed for more than 10 days. Multiple tests for bilirubin and liver function were normal during her hospitalization. Perhaps MP, a biological organism with an affinity for cholesterol, caused this phenomenon in her body. After 12 days of treatment, she was successfully discharged from the hospital (Figure 2).

A follow-up abdominal ultrasound showed no abnormalities on the sixth day after discharge. After her discharge, she continued to take prednisone tablets orally: 5 mg twice a day for 3 days, then changed to 5 mg once in the morning for 3 days, and finally changed to 2.5 mg once in the morning for 3 days before stopping the medication. The cough disappeared about 1 week after discharge, and there were no further digestive symptoms, such as abdominal pain or other complications. At 38 days after discharge, a follow-up examination of cytokines and an abdominal CT scan showed normal results. There had been no further abnormalities in blood sugar levels. The IgM antibody titer for MP was 4.79 COI, which had increased by more than four times.

## Discussion

The incidence rate of AP in children is 10–15 per 100,000, with a mortality rate of 1%–5% (4). Multiple risk factors can lead to childhood pancreatitis, such as genetic, obstructive, medicines, trauma, infection, metabolic issues, alcohol, and even endoscopic retrograde cholangiopancreatography. A diagnosis of AP requires two of the following three criteria: (1) abdominal pain consistent with pancreatitis; (2) serum amylase or lipase levels three or more times the upper limit of the normal range; and (3) consistent with pancreatitis on cross-sectional abdominal imaging findings, e.g., CT, magnetic resonance imaging, or transabdominal ultrasound (4, 5).

The diagnosis of AP and severe *M. pneumoniae* pneumonia (SMPP) in this 4-year-old girl was precise. The critical point is whether there is a causal relationship between them. This patient did not have alcohol-related factors, biliary diseases, congenital malformations, metabolic conditions, or other apparent conditions. It was most likely caused by infection or medication. Although multiple bacterial and viral infections can lead to pancreatitis, we excluded common bacteria, adenoviruses, influenza viruses, respiratory syncytial viruses, and coronaviruses through sputum culture and PCR. Unfortunately, we were unable to exclude all viruses. Common medications that cause pancreatitis include antiepileptics, notably valproic acid; the cancer drug asparaginase; and immunomodulators, such as thiopurines, mesalamine, and corticosteroids. Establishing causality for a particular drug as a risk factor for pancreatitis requires several lines of evidence, including whether there is an apparent temporal sequence, a typical latency period, recurrence of pancreatitis after a repeat challenge, or known mechanisms for developing pancreatitis with the drugs (6). Based on her current clinical evidence, infection and medicine were the most likely causes of AP. The excessive immune response caused by MP necessitates using glucocorticoids for treatment. However, glucocorticoids may also be the culprit in AP. After discussing and analyzing the case, we and her parents agreed with the monistic interpretation. The drugs she used, methylprednisolone sodium succinate, may also cause pancreatitis; however, the quality of the evidence is low (7). Finally, we used methylprednisone sodium succinate again and the relief effect of methylprednisolone to this girl excluded this argument. Therefore, based on her clinical symptoms, imaging findings, the positive nucleic acid test for MP, and elevated MP - IgM, we believe that the AP, in this case, was highly likely caused by MP infection.

The pathogenesis of extrapulmonary complications caused by MP has been explored, and three hypotheses have been proposed: (1) a direct attack by MP induces direct damage to local cells through the local action of bacterial lipoproteins; (2) changes in the immune regulation through the action of autoimmunity or immune complexes caused inflammatory reactions in extrapulmonary organs indirectly; and (3) vascular occlusion caused by direct or indirect action of MP (8). Some studies speculated that the second hypothesis leads to severe extrapulmonary complications. A dramatic increase in inflammatory cytokines and sound therapeutic effects of corticosteroids were noted in our case, supporting this speculation. Multiple cytokines can mediate immune inflammatory responses. Antigen components, such as toxins released by MP, can serve as pro-inflammatory factors, stimulate inflammatory reactions, and induce the production of cytokines, such as IL-2, Il-6, IL-17A, IFN-γ, and TNF, leading to inflammatory damage to tissues and organs (9).

Moreover, there are differences in the immune response patterns between general MPP and SMPP, which are reflected in serum cytokines (10). It was reported that IL-6, TNF, and IFN-γ significantly increased in children with MPP and were associated with the severity of the condition. These cytokines were further elevated in children with SMPP (11). The measured values of IL-2 in children with SMPP were higher than those in children with mild MPP; perhaps they can be used to distinguish between mild MPP and SMPP in children. These cytokines produced by immune inflammatory reactions indirectly cause damage to the pancreas, leading to the occurrence of pancreatitis (12). In recent years, IL-6 and IL-17A have become hot research topics, with several studies suggesting that they are involved in the process of AP and can be used in clinical practice to predict the severity of pancreatitis (13, 14). In this case, the girl experienced an increase in IL-2, IL-6, IL-17A, IFN-γ, and TNF during AP induced by MPP. After treatment, inflammatory cytokines showed varying degrees of decline. Hence, early monitored cytokine may be an early warning indicator for SMPP or extrapulmonary complications.

The transient blood glucose fluctuations in the girl may be due to hormonal effects or MP infection transiently damaging or inhibiting the endocrine system of the pancreas. In the past, there have been cases of MP infection with blood sugar fluctuations or even diabetes (15), but whether there is an inevitable causal relationship still needs further research to reveal the answer. In our case, it was considered a stress-induced hyperglycemia reaction. After using methylprednisolone again, we conducted rigorous blood sugar monitoring and found that blood sugar levels were within the normal range.

In the past 25 years, there have been a total of four cases of AP caused by MPP in children, of which two were misdiagnosed as appendicitis, indicating a high rate of misdiagnosis (Table 2).

**Table 2:** Cases of acute pancreatitis caused by *M. pneumoniae* in children in the past 25 years.

Cases	Sex/age (years)	Chief complaints	Is it misdiagnosed?	Other complications	*M. pneumoniae* detection	Treatment	Admission days
2 (15)	F/3	Vomit, fever, abdominal pain	Yes. Misdiagnosis as appendicitis	No	IgM	Macrolides	Unknown
1 (16)	F/6	Cough, sputum, epigastric pain, vomiting, fever, drowsy	No	Portal vein thrombus, pseudocyst	IgM	Gabexate mesylate, meropenem, clarithromycin	42
3 (17)	M/9	Mild fever, dry cough, anorexia, mild central abdominal pain, and occasional vomiting	Yes. Misdiagnosis as appendicular perforation	No	IgM	No antibiotic	Unknown
4 (18)	F/8	Cough and fever	No	No	IgM	CRAI + IV gabexate mesylate, meropenem	25
Current case	F/4	Cough and fever, abdominal pain	No	No	IgM + PCR	Azithromycin, erythromycin, clindamycin, methylprednisolone sodium succinate	12

CRAI, continuous regional arterial infusion.

The parents of this girl believed that early detection and consideration of AP caused by MPP and timely treatment were critical to the success of this treatment, which prevented further deterioration of the condition and the occurrence of other complications. This success was attributed to the communication and trust between doctors and patients.

In summary, in patients with MP infection and abdominal pain, AP should be considered in the differential diagnosis. Amylase, lipase, and pancreatic imaging should be performed. Inflammatory cytokines may mediate pancreatic damage.

## Data Availability

The datasets presented in this study can be found in online repositories. The names of the repository/repositories and accession number(s) can be found in the article/Supplementary Material.
